# Compliance of primary and secondary care public hospitals with standard practices for reprocessing and steam sterilization of reusable medical devices in Nepal: findings from nation-wide multicenter clustered audits

**DOI:** 10.1186/s12913-020-05788-0

**Published:** 2020-10-07

**Authors:** Gopal Panta, Ann K. Richardson, Ian C. Shaw, Patricia A. Coope

**Affiliations:** 1 Save the Children, Kathmandu, Nepal; 2 School of Health Sciences, University of Canterbury, Christchurch, New Zealand; 3 School of Physical and Chemical Sciences, University of Canterbury, Christchurch, New Zealand; 4 College of Education, Health and Human Development, University of Canterbury, Christchurch, New Zealand

**Keywords:** Medical devices, Reprocessing, Steam sterilization, Audits

## Abstract

**Background:**

Reusable medical devices in healthcare facilities are decontaminated and reprocessed following standard practices before each clinical procedure. Reprocessing of critical medical devices (those used for invasive clinical procedures) comprises several processes including sterilization, which provides the highest level of decontamination. Steam sterilization is the most used sterilization procedure across the globe. Noncompliance with standards addressing reprocessing of medical devices may lead to inadequate sterilization and thus increase the risk of person-to-person or environmental transmission of pathogens in healthcare facilities. We conducted nationwide multicenter clustered audits to understand the compliance of primary- and secondary-care public hospitals in Nepal with the standard practices for medical device reprocessing, including steam sterilization.

**Methods:**

We developed an audit tool to assess compliance of hospitals with the standard practices for medical device reprocessing including steam sterilization. Altogether, 189 medical device reprocessing cycles which included steam sterilization were assessed in 13 primary and secondary care public hospitals in Nepal using the audit tool. Percentage compliance was calculated for each standard practice. Mean percentage compliances were obtained for overall primary and secondary care hospitals and for each hospital type, specific hospital and process involved.

**Results:**

For all primary and secondary care hospitals in Nepal, the mean percentage compliance with the standard practices for medical device reprocessing including steam sterilization was 25.9% (95% CI 21.0–30.8%). The lower the level of care provided by the hospitals, the lower was the mean percentage compliance, and the difference in the means across the hospital types was statistically significant (*p* < 0.01). The mean percentage compliance of individual hospitals ranged from 14.7 to 46.0%. The hospitals had better compliance with the practices for cleaning of used devices and transport and storage of sterilized devices compared with the practices for other processes of the medical device reprocessing cycle.

**Conclusion:**

The primary and secondary care hospitals in Nepal had poor compliance with the standard practices for steam sterilization and reprocessing of medical devices. Interventions to improve compliance of the hospitals are immediately required to minimize the risks of person-to-person or environmental transmission of pathogens through inadequately reprocessed medical devices.

## Background

**Fig. 1:**
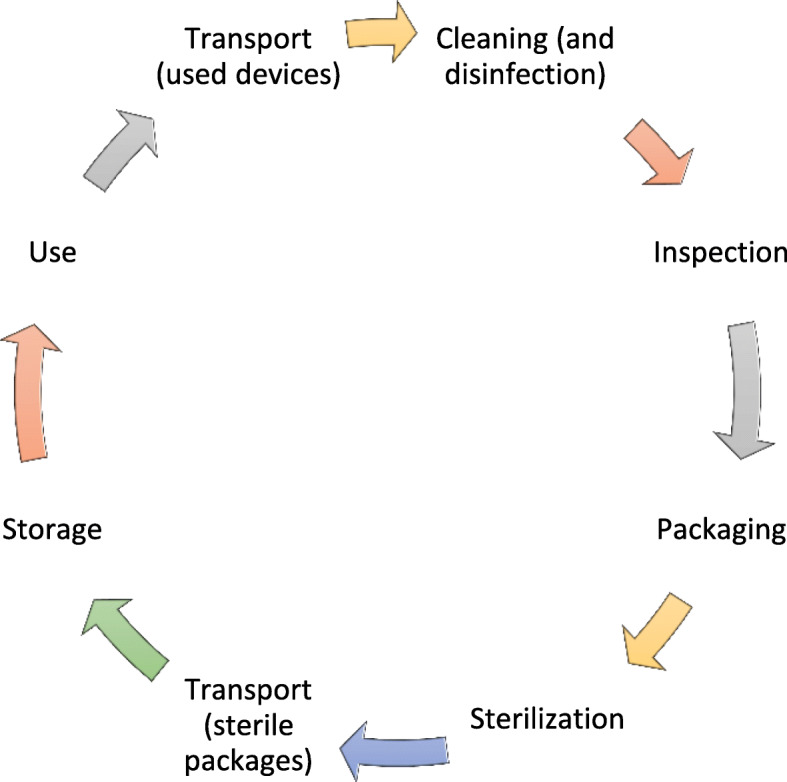
Medical device reprocessing cycle for a critical medical device [4]

Medical devices are used for a wide range of healthcare activities including prevention, diagnosis, treatment and monitoring of diseases or injuries. Many of these devices are reused several times after adequate decontamination and reprocessing. Medical devices used for invasive clinical procedures such as surgeries are categorized as critical medical devices and sterilized before reuse to prevent person-to-person or environmental transmission of pathogens [1]. Sterilization ensures the highest level of decontamination of the devices by making them free from any living microorganisms including spores which are the most resistant forms of microorganisms [2]. Steam sterilization (also known as moist-heat sterilization or autoclaving) is the most used technique for sterilizing reusable medical devices across the globe [3]. However, sterilization is not the only process used to free medical devices from viable microorganisms. Medical devices are subjected to a reprocessing cycle (Fig. 1) which comprises several processes including transport of used devices, cleaning, disinfection, inspection, packaging, sterilization, and transport and storage of sterilized devices [4]. The combination of steps that comprise the overall cleaning and sterilization process determines the effectiveness of the overall process. For example, if the initial cleaning step is ineffective and debris remains on the device, this will likely reduce the effectiveness of the steam sterilization process [5]. Various national/international guiding documents have recommended standard practices for each of these processes to achieve an internationally accepted Sterility Assurance Level (SAL) of critical medical devices. The accepted SAL of medical devices is 10^− 6^; i.e. the probability of getting a non-sterile medical device after subjecting it to a reprocessing cycle is 1 in a million [6, 7]. If any of the recommended practices are not followed, the risk of getting a non-sterile medical device after following the reprocessing cycle increases. Consequently, the risk of person-to-person or environmental transmission of pathogens through medical devices increases concomitantly.

It has been estimated that 7.1% (95% Confidence Interval (CI) 6.5–7.8%) and 10.2% (95% CI 9.0–13.0%) of hospitalized patients acquire healthcare-associated infections (HAIs) in developed and developing countries respectively, with surgical site infection (SSI) being the most common HAI in developing countries [8]. The pooled cumulative incidence of SSIs in low- and middle-income countries for the period of 1995–2008 has been reported to be 11.8 (95% CI 8.6–16.0) per 100 patients who had undergone surgical procedures [8]. Though scientific estimates for HAIs in Nepal are not available, some studies in tertiary care hospitals have shown SSI rates ranging from 2.7 to 23.0 per 100 patients [9–11]. Various studies have reported HAIs linked with inadequately sterilized medical devices in different places [12–16]. A recent review found steam sterilization failure rates ranging from 1.5 to 43.0% worldwide, with higher failure rates in developing countries [17].

Panta et al. previously reported a steam sterilization failure proportion of 69.8% (95% CI 44.4–87.0%) in primary and secondary care hospitals in Nepal [18]. To our knowledge, this proportion is the highest reported sterilization failure proportion in the world. Therefore, it is important to understand the extent of compliance of these hospitals with the standard practices for medical device reprocessing, including steam sterilization. Understanding the extent of compliance can help hospitals develop strategies and interventions to improve the practices and minimize the risks of transmission of pathogens through the reusable devices. A gap analysis of infection control practices in low- and middle-income countries indicated poor compliance of a tertiary-care teaching hospital in Nepal; where only 45% of the recommended practices for sterilization and disinfection were followed [19]. However, compliance of the primary and secondary care public hospitals in Nepal with the standard practices for medical device sterilization and reprocessing has not been studied comprehensively and gaps in this area have not been identified precisely.

We report findings of multicenter clustered audits which were carried out to assess compliance of the primary and secondary care public hospitals in Nepal with standard practices for medical device reprocessing and steam sterilization.

## Methods

### Audit tool: medical device reprocessing and steam sterilization

In Nepal, there is no nationally endorsed guiding document providing standard practices for medical device reprocessing or for steam sterilization. Therefore, first, we identified minimum essential practices [20] for medical device reprocessing and steam sterilization based on several published studies [21–29] and national/international guidelines and standards [30–42]. In this study, we use the term ‘standard medical device reprocessing practices’ or simply ‘standard practices’ for those minimal essential practices. The standards are prepared by an expert panel and are based on consensus [43, 44]. The criteria in the standards are used because in Nepal it is expected that the hospitals comply with the standards.

To assess the compliance of the primary and secondary care public hospitals with the standard practices for medical device reprocessing and steam sterilization, we developed an audit tool which included all standard practices as defined above. The process of development of the audit tool included a literature search in selected databases (Google, Google Scholar, Medline and CINAHL), formulation of a draft tool, further revision of the tool by experts [a public health expert from Nepal, a professor and physician working in the area of infection prevention and control in Nepal, a clinical nurse specialist working in infection prevention and control in a tertiary care hospital in New Zealand and supervisors of this study (AKR and ICS)], field-testing of the draft tool, and finalization of the tool (see Additional File 1). The tool comprised different sections related to medical device reprocessing with moist-heat sterilization. The sections in the tool were general, transport, cleaning and disinfection, inspection, packaging, steam sterilization (autoclaving), and transport and storage (Fig. 1). Each section included the basic elements required for medical devices reprocessing and steam sterilization in healthcare facilities.

### Sample design

The findings reported in this article are a part of a comprehensive observational study which comprised an estimation of steam sterilization failure proportions, an assessment of compliance of hospitals with standard medical device reprocessing practices, and a survey on knowledge and attitudes of healthcare workers towards sterilization and medical device reprocessing. The sample reported here was primarily developed for estimating steam sterilization failures in primary and secondary care public hospitals in Nepal and has been described in detail elsewhere [18].

We included three types of public hospitals in this study – zonal (secondary care), district (primary care) and district-level (primary care) hospitals. There were 10 zonal hospitals, 62 district hospitals and 16 district-level hospitals in Nepal [45]. Given the three types of hospitals with different attributes, we used a stratified design with three strata. Hospitals were sampled from within each stratum and simple proportional allocation of hospitals within each stratum was used. Each hospital represented a cluster of observations (the repeated sampling of the reprocessing cycle). Participation of hospitals in the study was voluntary.

### Sample size

We randomly sampled 2 zonal hospitals, 9 district hospitals and 2 district-level hospitals. The numbers of medical device reprocessing cycles to be audited in each hospital were 12, 15 and 15 for zonal, district and district-level hospitals, respectively. These numbers were primarily determined for estimating the proportion of steam sterilization failures in these hospitals and were based on the total number of hospitals within each category and a number of statistical assumptions including confidence level, margin of error required and impact of clustering [18]. The total number of medical device reprocessing cycles audited was 189 (Table 1).

**Table 1:** Sample sizes for auditing medical device reprocessing cycles in different hospital types

Hospital type	Number of hospitals	Randomly sampled hospitals	Consecutive reprocessing cycles audited in each hospital	Reprocessing cycles audited in each hospital type
Zonal hospital	10	2	12	24
District hospital	62	9	15	135
District-level hospital	16	2	15	30
**Total number of reprocessing cycles audited**				**189**

### Sample selection

To select hospitals within each hospital type, we used simple random sampling. Random sampling was carried out within Excel. For selection of zonal hospitals, each hospital was assigned a random number to four decimal places, between 0 and 1. Then, the hospitals were sorted in ascending order of random number and the first 2 hospitals in the list were selected into the sample. Two district-level hospitals were selected following the same approach as for the zonal hospitals. For district hospitals, we wanted the sample (i.e. nine hospitals) spread across the seven provinces, so a systematic random sampling method was chosen. A list of all district hospitals was made in order of province, with the hospitals randomly ordered within each province. The hospital where field-testing had been carried out was omitted from the whole process of sampling and therefore, nine hospitals had to be selected from the list of 61 hospitals. For this, one hospital was randomly chosen first within the range 1 to 61 and then every 7th hospital was selected.

Within a hospital, medical device reprocessing is a continuous process and the ‘population’ for the purpose of this study (i.e. total number of reprocessing cycles) was effectively infinite. It was impractical to select the reprocessing cycles randomly from such a population. Therefore, we audited a predetermined number (see Table 1) of consecutive medical device reprocessing cycles in each hospital.

### Data collection procedure

The audits were carried out by the researcher (GP) who was external to, and independent of, the hospital environments under study. All core processes of a medical device reprocessing (with steam sterilization) cycle were observed by the researcher and observations were recorded in the audit tool. The same audit process was repeated for all 189 medical device reprocessing cycles.

### Data management and analysis

A unique number was assigned to each hospital and recorded on each audit tool used in the study. Information from the audit tools was entered in a database (Excel spreadsheet) every day. After the completion of field work, data in the spreadsheets was imported to the IBM SPSS Statistics 24 software. Imported data sets were checked for errors and discrepancies. Identified errors and discrepancies were corrected by referring to the completed audit tools.

We performed descriptive analyses of information obtained from audits. Mean percentage compliance with standard reprocessing practices was obtained by calculating the mean of the percentage of standard practices followed for a reprocessing cycle by a hospital. Here, to calculate the percentage of standard practices followed, the numerator was the number of standard practices followed and the denominator was the number of applicable practices. Mean percentage compliance for each of the core processes of the reprocessing cycle was also calculated for each hospital type and for overall hospitals. The complex sample design was taken into account when performing these analyses.

One-way Analysis of Variance (ANOVA) was performed to determine the difference in the mean percentage compliance between the three hospital types. In addition, a pairwise multiple comparison test (*Tamhane’s T2*, a one-way ANOVA post hoc test) was performed to determine the difference in the means between each pair of hospital types [46].

## Results

Medical devices which were reprocessed in the hospitals included in this study had different designs including hollow, pin and box joints, channel, tubing and porous. Both metallic and non-metallic devices were reprocessed in the hospitals (Table 2). The processes of medical device reprocessing took place in a dirty to clean workflow for only 10.1% (95% CI 1.8–40.9%) of the reprocessing cycles.

**Table 2:** Percentages of reprocessing cycles including medical devices with different designs and materials

Design and material of reprocessed medical devices	Estimate (percentage of reprocessing cycles)	Standard Error	95% Confidence Interval	
			Lower	Upper
**Designs**^a^				
Solid, hollow	100.0%	0.0%	100.0%	100.0%
Pin and box joints	100.0%	0.0%	100.0%	100.0%
Channel, tubing	46.4%	5.0%	35.6%	57.6%
Porous	91.9%	3.4%	80.6%	96.9%
**Material**				
Metal	100.0%	0.0%	100.0%	100.0%
Non-metal	92.4%	3.4%	80.5%	97.3%

^a^ Examples of medical devices with different designs - Solid, hollow: bowl, dish, scalpel handle; Pin and box joints: scissors, forceps; Channel, tubing: urinary catheter, cannulated screws, dental hand piece; Porous: Cotton, gauze, linens

### Transport of used medical devices

In no reprocessing cycles were used medical devices transported to the decontamination area using an appropriate container (a rigid, durable, leak-proof container with a tight-fitting lid) [47]. However, all the containers used for transporting used medical devices were easy to clean and disinfect.

### Cleaning and disinfection

Medical devices were cleaned before sterilization for all the reprocessing cycles. Support staff (office assistants) were involved in the cleaning of medical devices for 98.4% (95% CI 88.3–99.8%) of the reprocessing cycles. Nursing staff were involved in the cleaning of medical devices for only 1.6% (95% CI 0.2–11.7%) of the reprocessing cycles. Medical devices were cleaned manually for all the reprocessing cycles.

The time between use and cleaning of medical devices ranged from about 20 min to about 2880 min (i.e. about 48 h). The estimated average time between use and cleaning of medical devices was about 298 min (95% CI 101–495). For an estimated 27.6% (95% CI 16.2–43.0%) of the reprocessing cycles, the time between use and cleaning of medical devices was about 60 min. For an estimated 19.3% (95% CI 10.4–33.0%) of the reprocessing cycles, the time between use and cleaning of medical devices was about 120 min.

Different cleaning agents, including disinfectant solutions, detergent/soap solutions and plain water, were used in different combinations for manual cleaning of medical devices (Table 3). Enzymatic cleaners were never used for cleaning the medical devices.

**Table 3:** Percentages of reprocessing cycles using different cleaning processes

Cleaning agents used	Estimate	Standard Error	95% Confidence Interval	
			Lower	Upper
Disinfectant solution → detergent/soap solution → plain water^a^	53.6%	10.8%	30.5%	75.3%
Disinfectant solution → detergent/soap solution^a^	9.3%	8.7%	1.0%	50.5%
Disinfectant solution → plain water^a^	18.8%	7.3%	7.4%	40.2%
Detergent/soap solution → plain water^a^	7.1%	5.0%	1.4%	29.6%
Plain water only	11.2%	6.5%	2.9%	35.1%

^a^ the agents were used for cleaning of medical devices in the given sequence

Though medical devices were cleaned manually before sterilization for all the reprocessing cycles, standard practices for cleaning were not always followed. Some practices, including cleaning of channels with brushes of appropriate size, were non-existent (Table 4).

**Table 4:** Percentages of reprocessing cycles following standard cleaning (and disinfection) practices

Standard practices	Estimate	Standard Error	95% Confidence Interval	
			Lower	Upper
Used medical devices are soaked in or sprayed with water before cleaning, to prevent drying	81.7% ^a^	7.9%	57.9%	93.5%
Cleaning is done in a separate area from where the instrument will be used (i.e., designated dirty area)	38.1%	11.5%	17.3%	64.5%
Medical devices are pre-disinfected before cleaning (e.g. with hypochlorite solution)	81.7%	7.9%	57.9%	93.5%
Medical devices are opened/dismantled for cleaning purpose	76.4%	10.7%	46.4%	92.4%
Medical devices are submerged in water while washing them manually using a brush	1.0%	1.0%	0.1%	7.6%
For instruments with channels, all channels are cleaned using cleaning brushes of appropriate size	0.0%	–	–	–
Cleaning brushes are single use (disposable) items	0.0%	–	–	–
After completion of cleaning, reusable brushes are cleaned and either high level disinfected or sterilized	0.0%	–	–	–
Instruments are rinsed thoroughly with water after cleaning	86.6%	9.0%	53.3%	97.3%
Medical devices are dried with low-linting (disposable or reusable) towels immediately after rinsing	19.9%	8.1%	7.4%	43.4%
Enzymatic cleaner, detergent, and/or disinfectant are used according to manufacturer’s instructions	68.3%	12.4%	37.7%	88.5%

^a^ medical devices were soaked in hypochlorite solution instead of plain water

Gloves were the only personal protective equipment (PPE) used by staff during cleaning for most of the reprocessing cycles. Eye protection, face masks and protective clothing were rarely used (Table 5).

**Table 5:** Percentages of reprocessing cycles for which staff used PPE during cleaning

	Estimate	Standard Error	95% Confidence Interval	
			Lower	Upper
Eye protection	1.1%	1.0%	0.1%	8.0%
Gloves	97.9%	1.1%	93.6%	99.3%
Protective clothing	4.8%	4.4%	0.6%	30.5%
Facemask	6.4%	5.4%	0.9%	33.7%

### Inspection

Medical devices were inspected after cleaning for 30.5% (95% CI 15.6–50.9%) of the reprocessing cycles. However, an illuminated magnifier was not used to inspect instruments after cleaning in any of the reprocessing cycles.

### Packaging

Linen was used as the wrapping material for all of the reprocessing cycles, which included wrapped medical devices in the sterilization load. The envelope fold wrapping technique was used at all times when medical devices were wrapped. Hinged devices were opened, or devices were dissembled while packing them for only 1.2% (95% CI 0.2–8.1%) of the reprocessing cycles. For 28.8% (95% CI 12.5–53.5%) of the reprocessing cycles, packages were labelled with the date of sterilization. Similarly, for 8.0% (95% CI 0.9–45.0%) of the cycles, packages were labelled with the expiry date. For none of the reprocessing cycles, were packages labelled with the sterilizer used and the cycle or load number.

### Sterilization (autoclaving)

Support staff (office assistants) carried out the autoclaving process for 97.0% (95% CI 87.5–99.3%) of the reprocessing cycles whereas nursing staff carried out the process for only 3.0% (95% CI 0.7–12.5%) of the reprocessing cycles. For none of the autoclave cycles, were parameters including cycle/load number, operator, sterilization date and time, pressure, temperature and holding period recorded. Autoclave tape was used for some of the autoclave cycles (Table 6). However, biological and chemical indicators were not used in any of the autoclave cycles.

**Table 6:** Percentages of reprocessing cycles following standard autoclaving practices

Standard practices	Estimate	Standard Error	95% Confidence Interval	
			Lower	Upper
Timer is used to monitor holding period of the manually controlled autoclave cycle	6.4%	2.8%	2.4%	16.1%
Holding period of the autoclave cycle starts when the pressure gauze shows the reading of required pressure (e.g.15 lbs)	18.2%	8.0%	6.3%	42.4%
Indicators used for monitoring sterilization process				
Autoclave tape	48.7%	9.0%	29.8%	68.0%
- Result of autoclave tape is recorded	0.0%	–	–	–
- Autoclave tape is used on the outside of each wrapped package (for the loads where autoclave tape is used)	79.4%	7.8%	57.0%	91.8%
Sterilized packs are intact and dry	10.8%	5.1%	3.6%	28.5%

### Transport and storage

Standard practices for transport and storage of sterilized medical devices were not followed for most of the reprocessing cycles (Table 7).

**Table 7:** Percentages of reprocessing cycles following standard transport and storage practices

Standard practices	Estimate	Standard Error	95% Confidence Interval	
			Lower	Upper
Sterilized packages are checked for integrity, and compromised packages are repackaged and re-sterilized before use	0.0%	–	–	–
Sterilized items are transported and delivered in a dry and clean container	47.2%	9.4%	27.8%	67.5%
Sterilized packages are allowed to cool down to room temperature before storage	89.1%	6.8%	63.3%	97.5%
A separate area is allocated for storage of sterilized medical devices	40.9%	6.7%	27.1%	56.3%
Sterilized packages are stored and distributed according to “the first one to enter is the first one to leave”	25.1%	8.9%	10.4%	49.1%
The area for storing sterilized packages is well-ventilated and provides protection against dust, moisture, insects, and temperature and humidity extremes	31.5%	16.5%	7.8%	71.6%

### Mean percentage compliance

The mean percentage compliance for all primary and secondary care hospitals was 25.9% (95% CI 21.0–30.8%). The higher the hospital level, the higher was the mean percentage compliance with the standard reprocessing practices (Table 8). The difference in the means was found to be statistically significant (*p* < 0.01). The means were also statistically significantly different (*p* < 0.01) between each pair of hospital types (i.e. between zonal hospital and district hospital, between district hospital and district-level hospital, and between district-level hospital and zonal hospital).

**Table 8:** Mean percentage compliance with standard reprocessing practices for hospital types

Hospital type	Percentage Estimate	Standard Error	95% Confidence Interval	
			Lower	Upper
Zonal hospital	32.0%	0.1%	31.8%	32.1%
District hospital	26.6%	3.0%	19.9%	33.4%
District-level Hospital	19.6%	0.1%	19.4%	19.7%

Comparatively, hospitals were more compliant with recommendations for cleaning and disinfection, and transport and storage of medical devices. However, compliance with these processes was below 50% (Table 9).

**Table 9:** Mean percentage compliance for core processes of a reprocessing cycle

Core processes of reprocessing cycle	Hospital types	Percentage Compliance	Standard Error	95% Confidence Interval	
				Lower	Upper
**Transport of used devices**	**All hospitals**	**26.1%**	**5.6%**	**13.7%**	**38.5%**
	Zonal hospitals	27.3%	17.7%	0.0%	66.8%
	District hospitals	23.4%	6.4%	9.0%	37.7%
	District-level hospitals	35.7%	7.2%	19.8%	51.7%
**Cleaning and disinfection**	**All hospitals**	**45.8%**	**2.2%**	**40.8%**	**50.7%**
	Zonal hospitals	54.6%	3.2%	47.5%	61.8%
	District hospitals	46.5%	3.0%	39.9%	53.2%
	District-level hospitals	37.8%	1.5%	34.5%	41.0%
**Inspection and packaging**	**All hospitals**	**10.9%**	**2.3%**	**5.7%**	**16.1%**
	Zonal hospitals	19.8%	6.2%	6.0%	33.5%
	District hospitals	12.3%	3.1%	5.4%	19.2%
	District-level hospitals	0.0%	0.0%	0.0%	0.0%
**Sterilization (autoclaving)**	**All hospitals**	**9.0%**	**1.5%**	**5.7%**	**12.3%**
	Zonal hospitals	11.1%	0.3%	10.5%	11.6%
	District hospitals	10.2%	1.9%	6.0%	14.4%
	District-level hospitals	2.9%	2.8%	0.0%	9.1%
**Transport and storage**	**All hospitals**	**39.3%**	**5.5%**	**27.0%**	**51.6%**
	Zonal hospitals	43.9%	4.2%	34.5%	53.3%
	District hospitals	37.9%	7.7%	20.7%	55.1%
	District-level hospitals	42.3%	2.2%	37.3%	47.2%

**Fig. 2:**
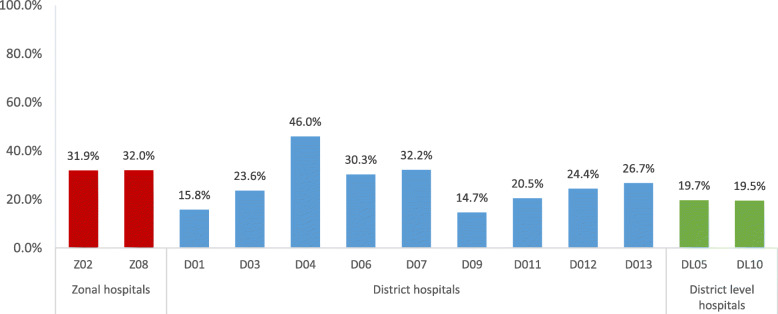
The mean percentage compliance (for each hospital) with standard practices for core processes of reprocessing cycle

Mean percentage compliances for the two zonal hospitals were similar. The percentage compliances for district hospitals ranged from 14.7 to 46.0%, showing considerable variation in practices across the hospitals. The two district level hospitals had similar average compliances (Fig. 2).

## Discussion

The mean percentage compliance with standard practices for the reprocessing of reusable medical devices achieved by all the primary and secondary care public hospitals in Nepal was only 25.9% (95% CI 21.0–30.8%). There is no standard cut-off value for percentage compliance with these practices. A high value of the percentage compliance indicates that most of the standard practices for medical device reprocessing are followed by the hospitals. Compliance with the standard practices helps hospitals ensure safety, reliability and quality of medical device reprocessing [48]. Therefore, it is important for the hospitals to comply with the standard practices for ensuring the required SAL of medical devices. In this sense, the compliance of the primary and secondary care hospitals in Nepal with the standard reprocessing practices is poor. The mean percentage compliances for each process of the reprocessing cycle were less than 50%. These findings are in line with the high proportion of steam sterilization failures (reported elsewhere) in these hospitals [18]. One factor that may have led to overestimation of the mean percentage compliance is the presence of the researcher (auditor) during the reprocessing activities. The staff involved in medical device reprocessing may have become more attentive due to the presence of the researcher and hence, they could have performed the reprocessing activities more carefully on the days when the researcher was present than on ‘normal’ days.

The reasons for the poor compliances are not clearly understood. We previously reported that more than 50% of these hospitals did not have dedicated space for reprocessing of medical devices, and only one of the thirteen hospitals had sterilization procedure manuals or flow charts. Similarly, only one hospital had spare parts for the sterilization equipment and none of the hospitals had equipment maintenance records. Moreover, only 1 to 3 staff were assigned for medical device reprocessing in these hospitals [18]. In this study, we found that, for more than 97% of the reprocessing cycles crucial processes, such as cleaning and autoclaving, were carried out by support staff (i.e. office assistants). The level of education of support staff is very poor i.e. either completion of some years in school or no formal education. These findings indicate that there could be several reasons for poor compliance related to management and support processes including infrastructure, human resources, equipment, guiding documents, steering, performance monitoring and documentation. As the higher-level hospitals, compared with the lower-level hospitals, should have better management and support processes, the higher average percentage compliance in these hospitals is to be expected.

About 92% of the reprocessing cycles had sterilization loads with porous items such as linen and more than 46% of the cycles had loads including items with channels or tubing, such as dental hand pieces and laparoscopic sheaths. Air in all cavities and spaces within such medical devices needs to be replaced with steam for proper sterilization. Air removal is more difficult with such items and active air removal is usually recommended for ensuring the attainment of sterilizing conditions [4, 40]. None of the steam sterilization processes used by primary and secondary care hospitals in Nepal had an active air removal process such as pre-vacuuming [18]. No specific sterilization processes were designated for medical devices having specific designs, and devices with different designs were included in a single load. Such practice in the absence of an active air removal process is detrimental to the achievement of efficient sterilizing conditions within the load.

Safe transportation of used medical devices is important to minimize microbial contamination of the surrounding environment, and also to minimize the risk of device-associated infection among healthcare workers and patients. A rigid, durable, leak-proof container with a tight-fitting lid is recommended for transportation of used medical devices to the decontamination area [47]. However, for all of the reprocessing cycles in the hospitals in Nepal, used medical devices were either transported in an inappropriate container or transported without using a container. Such an inappropriate handling practice may be putting healthcare workers and patients at risk of injuries and/or exposure to microorganisms.

Cleaning was done in a designated dirty area for only 38.1% of the reprocessing cycles. Cleaning of medical devices in areas where other activities such as hand washing, dish washing, food preparation and drinking are performed, poses a risk of contamination of other areas and thus increases the risk of transmission of microorganisms to healthcare workers and patients. The risk of transmission of microorganisms was further amplified by the practice of cleaning medical devices without submerging them in water. Washing medical devices without submerging them in water may create splashes and aerosols, which might not only transmit microorganisms, but also can also increase inhalation of disinfectant by the cleaners and increase contact of mucous membranes (e.g. mucous membranes of eyes and mouth) with the disinfectant.

The risk of infection among cleaning staff was further increased by very poor compliance with the recommended use of PPE. Though gloves were used during cleaning for most of the reprocessing cycles, use of eye protection, protective clothing and facemasks during the cleaning process was rare. A study conducted in one of the largest hospitals in Nepal found that 20.9% of “non-professional staff”, 19.2% of nurses, 5.6% of laboratory workers and 3.1% of doctors had evidence of past or present HBV infection [49]**.** The authors of the study claimed that higher occurrence of HBV among “non-professional staff” and nurses was because of their involvement in the cleaning of medical devices without proper measures to protect themselves and the lack of adequate HBV vaccination. The findings of our study support the claim made by these authors.

Medical devices were cleaned manually for all of the reprocessing cycles in all of the hospitals. Automated washers are commonly used in many countries for cleaning of reusable medical devices, but studies have found that both manual and automated cleaning processes are effective in reducing the microbial load on medical devices if executed properly [50, 51]. Manual cleaning processes are more prone to human error compared to automated processes. Ofstead et al. (2010) found adherence to endoscope reprocessing guidelines for 1.4% of endoscopes reprocessed manually, and for 75.4% of endoscopes reprocessed with an automated endoscope cleaner and reprocessor [52]. We found variations in manual cleaning processes in the hospitals of Nepal as well, the process varying from single-step cleaning using plain water to three-step cleaning using disinfectant, detergent/soap and plain water.

For about 82% of the reprocessing cycles, the cleaning process included pre-soaking of medical devices in a hypochlorite solution. In the absence of proper and consistent use of PPEs, the practice of pre-soaking might have provided some protection to the staff handling used medical devices, however, this practice could have deterred staff from the proper and consistent use of PPEs. Recent guidelines recommend not pre-soaking medical devices in a disinfectant before cleaning [4]. The reasons behind this recommendation are corrosion of the devices due to disinfectants, inactivation of disinfectant by blood and body fluids, risks to health-care workers while transporting the soaked items and possible contribution to the development of antimicrobial resistance to the disinfectants [4]. However, in the case of Nepal, if pre-soaking in hypochlorite solution were to be avoided, the practices of cleaning medical devices immediately after the procedure (usually within 1 h) will become crucial for effective cleaning of medical devices and the prevention of formation of biofilms on medical devices [53].

Cleaning of medical devices is a critical step for reprocessing of medical devices, as it significantly reduces bioburden on the surfaces of medical devices [51]. However, this is not as simple as it may appear. Staff responsible for cleaning of medical devices need to have a clear understanding of microorganisms and the importance of cleaning in medical device reprocessing. Seavey (2009) highlights the need for educating staff involved in reprocessing activities at least in the areas of basic medical terminology, human anatomy and physiology, microbiology, infection prevention and control, regulations and standards, surgical instruments, and all processes of reprocessing cycles [54]. In an ideal context, monitoring of cleaning processes using a validated scientific monitoring technique is recommended for ensuring adequate cleaning of medical devices [55]. However, support staff (office assistants) with very poor education level were involved in the cleaning of medical devices for almost all (98.4%) of the reprocessing cycles in the primary and secondary care hospitals. A required level of cleaning of medical devices is unlikely to be achieved without having properly trained and educated staff undertaking the reprocessing of medical devices.

For all the reprocessing cycles sterilizing wrapped medical devices, linens were used as the wrapping material. Previous studies have demonstrated the effectiveness of linens in maintaining sterility of wrapped medical packages [56, 57]. Currently, there are various options available for packaging of medical devices including rigid containers, peel pouches (plastic and/or paper), and woven and nonwoven wrapping materials. Based on cost-effectiveness and suitability, such options also need to be explored and used by hospitals for continuous improvement in medical device reprocessing.

Orientation and loading of channeled medical devices inside each medical device package and that of the packages in each sterilization load could not be recorded in this study. However, it is important to ensure that the packages are loaded ensuring adequate circulation of steam inside each of the packages and channeled and hollow devices are positioned inside the packages in such a way that accumulation of condensate inside such devices is avoided [58].

Most of the standard practices for steam sterilization (autoclaving) were not followed for most of the reprocessing cycles. No chemical or biological indicators were used to monitor the effectiveness of sterilization, except for the use of indicator tape for fewer than 50% of the reprocessing cycles. Indeed, autoclave tapes do not measure the effectiveness of autoclave cycles; they only indicate an exposure of a package to a heat/pressure-based sterilization process [59]. Additionally, none of the sterilization cycles had variable parameters (time, temperature and pressure) recorded. Though all the autoclave cycles were manually operated, timers were used to monitor sterilization holding periods for only a small proportion (i.e. 6.4%) of autoclave cycles. This showed that medical devices were being reused without having concrete evidence of their sterility. Information such as load number, operator, and sterilization date and time were also not recorded. In the case of an incident (such as surgical site infection) likely to be associated with reusable medical devices, it would be difficult to trace the sterilization load, person sterilizing the load, or the date and time of sterilization. This indicated that it was unlikely that the possible source of infection would be identified, thus preventing correction of faulty practices.

For the majority of the reprocessing cycles, sterilized packages were wet or contained moisture. The wet sterilized packages could have been associated with one or more factors including quality of packaging material, packaging technique, loading technique, sterilization process, sterilizer, steam quality and storage area [60]. Moisture can facilitate the entrance of microorganisms to the sterilized packages. In general, wet sterilized packages are considered as contaminated, and should be re-sterilized before use, and wet sterilized porous loads such as textiles can be even more problematic [61]. Some studies conducted in Nepal have shown that different microorganisms including *Staphylococcus aureus*, *Micrococcus* spp., coagulase-negative staphylococci, *Bacillus* spp., *Pseudomonas* spp., *Acinetobacter* spp. and yeasts exist in hospital indoor environments [62, 63]. In these settings where sterile storage conditions are not controlled, the chances of contamination of wet packages with microorganisms could be high. None of the wet sterilized packages were subjected to re-sterilization in the hospitals in Nepal. There is a need for a thorough assessment to establish the causes of wet sterilized packages to formulate recommendations for solving the problem.

The absence of routine inspection of packages after sterilization for integrity was observed in all of the hospitals. The absence of inspection of sterilized packages could also be linked with the practice of not re-sterilizing wet sterilized packages discussed above. There were gaps in transportation and storage of sterilized packages which did not favor long-term sterility of medical devices, which was further compromised by wetness of sterilized packages.

The findings of this study may not be able to be generalized to tertiary care public hospitals (i.e. central hospitals) and private hospitals in Nepal as these hospitals were not included in the study. The study also did not cover smaller public and private healthcare facilities such as primary health centres, health posts, private outpatient clinics and private dental clinics. However, the findings of this study can be useful for the improvement of medical device reprocessing in these healthcare facilities as well.

## Conclusion

To our knowledge, this study is the first of its kind in Nepal. This study provides an overall picture and baseline information on compliance of primary and secondary care hospitals in Nepal with the standard practices for steam sterilization and reprocessing of reusable medical devices. The compliance of the hospitals was found to be poor for all the processes of the reprocessing cycle, the overall compliance rate being lower with the lower-level hospitals. Adequate infrastructure, proper guiding documents, training of healthcare workers, adequate PPE, availability of essential materials and regular monitoring could help the hospitals improve the current situation of medical device reprocessing. Such an improvement could ultimately help to minimize the risk of transmission of pathogens associated with the reusable medical devices. The audit tool developed by us can be useful in other low- and middle-income countries to assess the compliance of the hospitals with standard medical device reprocessing practices.

## Supplementary information

**Additional file 1.** Audit tool for medical device reprocessing and steam sterilization practices

## Data Availability

All data generated or analysed during this study are included in this published article and its supplementary information files.

## Abbreviations

HAI: Healthcare Associated Infection
HBV: Hepatitis B Virus
PPE: Personal Protective Equipment
SAL: Sterility Assurance Level
SSI: Surgical Site Infection

## References

[1] Spaulding EH (1968). Chemical disinfection of medical and surgical materials. Disinfection, Sterilization and Preservation.

[2] Perkins JJ (1956). Principles and methods of sterilization.

[3] Rutala WA, Weber DJ (1999). Infection control: the role of disinfection and sterilization. J Hosp Infect..

[4] World Health Organization (WHO). Decontamination and reprocessing of medical devices for healthcare facilities. 2016. http://apps.who.int/iris/bitstream/10665/250232/1/9789241549851-eng.pdf. Accessed 17 Dec 2017.

[5] Miller CH (1993). Cleaning, sterilization and disinfection: basics of microbial killing for infection control. J Am Dent Assoc.

[6] Denyer S, Hodges N, Denyer SP, Hodges NA, Gorman SP (2004). Sterilization procedures and sterility assurance. Hugo and Russell's pharmaceutical microbiology.

[7] Allison DG (1999). A review: taking the sterile out of sterility. J Appl Microbiol.

[8] WHO. Report on the burden of endemic health care-associated infection worldwide. 2011. http://apps.who.int/iris/bitstream/10665/80135/1/9789241501507_eng.pdf. Accessed 7 Jul 2016.

[9] Giri BR, Pant HP, Shankar PR, Sreeramareddy CT, Sen PK (2008). Surgical site infection and antibiotics use pattern in a tertiary care hospital in Nepal. J Pak Med Assoc.

[10] Giri S, Kandel BP, Pant S, Lakhey PJ, Singh YP, Vaidya P (2013). Risk factors for surgical site infections in abdominal surgery: a study in Nepal. Surg Infect.

[11] Shrestha S, Wenju P, Shrestha R, Karmacharya RM (2016). Incidence and risk factors of surgical site infections in Kathmandu university hospital, Kavre. Nepal Kathmandu University Medical Journal.

[12] Esel D, Doganay M, Bozdemir N, Yildiz O, Tezcaner T, Sumerkan B, Aygen B, Selcuklu A (2002). Polymicrobial ventriculitis and evaluation of an outbreak in a surgical intensive care unit due to inadequate sterilization. J Hosp Infect.

[13] Dancer SJ, Stewart M, Coulombe C, Gregori A, Virdi M (2012). Surgical site infections linked to contaminated surgical instruments. J Hosp Infect..

[14] Tosh PK, Disbot M, Duffy JM, Boom ML, Heseltine G, Srinivasan A, Gould CV (2011). Berrı’os-Torres SI. Outbreak of *Pseudomonas aeruginosa* surgical site infections after arthroscopic procedures: Texas, 2009. Infect Control Hosp Epidemiol.

[15] Hildy M, Brown-Elliott BA, Douglas M, Curry J, Cecile T, Yansheng Z, Wallace RJ (2002). An outbreak of *Mycobacterium chelonae* infection following liposuction. Clin Infect Dis.

[16] Lu WP, Lin GX, Shi S, Dong JH (2012). Simultaneously high prevalence of hepatitis B and C virus infection in a population in Putian County. China J Clin Microbiol.

[17] Panta G, Richardson AK, Shaw IC (2019). Effectiveness of autoclaving in sterilizing reusable medical devices in healthcare facilities. J Infect Dev Ctries.

[18] Panta G, Richardson AK, Shaw IC, Chambers S, Coope PA (2019). Effectiveness of steam sterilization of reusable medical devices in primary and secondary care public hospitals in Nepal and factors associated with ineffective sterilization: A nation-wide cross-sectional study. PloS One.

[19] Weinshel K, Dramowski A, Hajdu Á, Jacob S, Khanal B, Zoltán M, Mougkou K, Phukan C, Staneloni MI, Singh N (2015). Gap analysis of infection control practices in low-and middle-income countries. Infect Control Hosp Epidemiol.

[20] Benjamin A (2008). Audit: how to do it in practice. BMJ..

[21] Bagg J, Smith AJ, Hurrell D, McHugh S, Irvine G (2007). Pre-sterilisation cleaning of re-usable instruments in general dental practice. Br Dent J.

[22] Bonetti D, Young L, Black I, Cassie H, Ramsay CR, Clarkson J (2009). Can't do it, won't do it! Developing a theoretically framed intervention to encourage better decontamination practice in Scottish dental practices. Implement Sci.

[23] Cooper T, Tait J, Bingham P (2003). Decontamination in primary care: development and implementation of a quality improvement programme using audit. Br J Infect Contr.

[24] Danchaivijitr S (2005). Development of quality indicators for sterilization practices of the central sterile supply department. J Med Assoc Thail.

[25] Finn L, Crook S (1998). Minor surgery in general practice-setting the standards. J Public Health.

[26] Matsuda JK, Grinbaum RS, Davidowicz H (2011). The assessment of infection control in dental practices in the municipality of São Paulo. Braz J Infect Dis.

[27] McNally O, Thompson IM, McIlvenny G, Smyth ETM, McBride N, MacAuley D (2001). Sterilization and disinfection in general practice within university health services. J Hosp Infect..

[28] Smith AJ, Bagg J, Hurrell D, McHugh S (2007). Sterilisation of re-usable instruments in general dental practice. Br Dent J.

[29] Smith AJ, Hurrell D, Bagg J, McHugh S, Mathewson H, Henry M (2007). (b). A method for surveying instrument decontamination procedures in general dental practice. Br Dent J.

[30] Acosta-Gnass SI, Stempliuk VDA. Sterilization manual for health centers. 2009. https://www.medbox.org/preview/5255d6fb-b598-4b60-b19a-02b60e695ecc/doc.pdf. Accessed 4 Feb 2015.

[31] Centers for Disease Control and Prevention. Infection prevention checklist for outpatient settings: Minimum expectations for safe care. 2014. https://www.cdc.gov/hai/settings/outpatient/outpatient-care-guidelines.html. Accessed 2 Feb 2015.

[32] Centers for Medicare and Medicaid Services. Exhibit 351: Ambulatory Surgical Center (ASC) infection control surveyor worksheet. 2013. https://www.cms.gov/Regulations-and-Guidance/Guidance/Manuals/downloads/som107_exhibit_351.pdf. Accessed 12 Mar 2015.

[33] Centers for Medicare and Medicaid Services. Hospital infection control worksheet. 2015. https://www.cms.gov/medicare/provider-enrollment-and-certification/surveycertificationgeninfo/downloads/survey-and-cert-letter-15-12-attachment-1.pdf. Accessed 25 Jun 2015.

[34] International Organization for Standardization (2006). Sterilization of health care products - moist heat - part 1: requirements for the development, validation and routine control of a sterilization process for medical devices (ISO 17665-1:2006 E).

[35] International Organization for Standardization (2009). Sterilization of health care products - moist heat - part 2: guidance on the application of ISO 17665-1 (ISO/TS 17665–2:2009 E).

[36] International Organization for Standardization (2013). Sterilization of health care products - moist heat - part 3: guidance on the designation of a medical device to a product family and processing category for steam sterilization ( ISO/TS 17665–3:2013 E).

[37] National Health Training Center - Ministry of Health and Population - Government of Nepal (2015). Infection prevention and healthcare waste management training - handbook for participants.

[38] National Health Training Center - Ministry of Health and Population - Government of Nepal (2015). Reference manual for infection prevention and healthcare waste management.

[39] Provincial Infectious Diseases Advisory Committee - Public Health Ontario. Best practices in cleaning, disinfection and sterilization of medical equipment/devices in all health care settings. 2013. http://www.publichealthontario.ca/en/eRepository/PIDAC_Cleaning_Disinfection_and_Sterilization_2013.pdf. Accessed 25 Jun 2015.

[40] Rutala WA, Weber DJ, Healthcare Infection Control Practices Advisory Committee. Guideline for disinfection and sterilization in healthcare facilities. 2008. https://www.cdc.gov/hicpac/pdf/guidelines/Disinfection_Nov_2008.pdf. Accessed 9 Feb 2015.

[41] Scottish Dental Clinical Effectiveness Programme. Cleaning of dental instruments - dental clinical guidance. 2014. http://www.sdcep.org.uk/wp-content/uploads/2016/03/SDCEP_Cleaning_of_Dental_Instruments_2nd_Edition_Jan2016.pdf. Accessed 5 Sep 2015.

[42] Standards Australia, & Standards New Zealand. Office-based health care facilities - reprocessing of reusable medical and surgical instruments and equipment, and maintenance of the associated environment (AS/NZS 4815:2006). 2006. https://shop.standards.govt.nz/catalog/4815%3A2006%28AS%7CNZS%29/view?client=html5. Accessed 3 Mar 2015.

[43] International Organization for Standardization. Consumers and standards: Partnership for a better world. 2020. https://www.iso.org/sites/ConsumersStandards/1_standards.html. Accessed 29 Aug 2020.

[44] International Organization for Standardization. Standardization and related activities - General vocabulary. 2004. https://isotc.iso.org/livelink/livelink/fetch/2000/2122/4230450/8389141/ISO_IEC_Guide_2_2004_%28Multilingual%29_-_Standardization_and_related_activities_%2D%2D_General_vocabulary.pdf?nodeid=8387841&vernum=-2. Accessed 2 Sep 2020.

[45] Department of Health Services—Ministry of Health and Population—Government of Nepal. Annual report 2013/2014. http://dohs.gov.np/wp-content/uploads/2014/04/Annual_Report_2070_71.pdf. Accessed 17 Aug 2015.

[46] IBM Knowledge Center. One-way ANOVA post hoc tests. 2017. https://www.ibm.com/support/knowledgecenter/en/SSLVMB_23.0.0/spss/base/idh_onew_post.html. Accessed 13 Dec, 2017.

[47] Miller CH, Palenik CJ, Block SS (2001). Sterilization, disinfection, and asepsis in dentistry. Disinfection, sterilization, and preservation.

[48] Bancroft R, Walker JT (2014). The role of standards in decontamination. Decontamination in hospitals and healthcare.

[49] Shrestha SK, Bhattarai MD (2006). Study of hepatitis B among different categories of health care workers. J Coll Physicians Surg Pak.

[50] Alfa MJ, Olson N, DeGagne P (2006). Automated washing with the reliance endoscope processing system and its equivalence to optimal manual cleaning. Am J Infect Control.

[51] de Souza ES, dos Santos SG, de Resende Stoianoff MA, de Oliveira AC (2015). Analysis of microbial load on surgical instruments after clinical use and following manual and automated cleaning. Am J Infect Control.

[52] Ofstead CL, Wetzler HP, Snyder AK, Horton RA (2010). Endoscope reprocessing methods: a prospective study on the impact of human factors and automation. Gastroenterol Nurs.

[53] Roberts CG (2013). The role of biofilms in reprocessing medical devices. Am J Infect Control.

[54] Seavey R (2009). The need for educated staff in sterile processing - patient safety depends on it. Perioperative Nursing Clinics.

[55] Alfa MJ (2013). Monitoring and improving the effectiveness of cleaning medical and surgical devices. Am J Infect Control.

[56] Barrett R, Stevens J, Taranter J (2003). A shelf-life trial: examining the efficacy of event related sterility principles and its implications for nursing practice. Aust J Adv Nurs.

[57] Bhumisirikul W, Bhumisirikul P, Pongchairerks P (2003). Long-term storage of small surgical instruments in autoclaved packages. Asian J Surg.

[58] Hoyos JVDG, van Wezel RAC, van Doornmalen HWJM (2015). Case study on the orientation of phaco hand pieces during steam sterilization processes. J Hos Infect.

[59] Proietti RM, Reichert M, Young JH (1997). Sterilization process monitoring: chemical indicators. Sterilization technology for the healthcare facility.

[60] Basu D (2017). Reason behind wet pack after steam sterilization and its consequences: an overview from central sterile supply department of a cancer center in eastern India. J Infect Public Heal.

[61] Huys J (2010). Sterilization of medical supplies by steam: general theory.

[62] Pradhan S, Shrestha C (2013). Microbiological surveillance of hospital environment in a medical College Hospital in Kathmandu, Nepal. Int J Infect Microbiol.

[63] Sapkota B, Gupta GK, Shrestha SK, Pradhan A, Karki P, Thapa A (2016). Microbiological burden in air culture at various units of a tertiary care government hospital in Nepal. Australas Med J.

