# Supplementary material for: Compliance of primary and secondary care public hospitals with standard practices for reprocessing and steam sterilization of reusable medical devices in Nepal: findings from nation-wide multicenter clustered audits
Source: BMC Health Serv Res. 2020 Oct 7;20:923. doi: 10.1186/s12913-020-05788-0 (PMC7542764; doi:10.1186/s12913-020-05788-0)
Supplement: Supplementary file 1 — Additional file 1. Audit tool for medical device reprocessing and steam sterilization practices [file 12913_2020_5788_MOESM1_ESM.docx]

**AUDIT TOOL FOR MEDICAL DEVICE REPROCESSING AND STEAM STERILIZATION PRACTICES**

**Hospital No: _____ Date: _ _/_ _/_ _ _ _ Observation No: ___/___**

**AA. GENERAL**

| **S.No.** | **Check Points** | **Yes** | **No** | **NA** | **Comments** |
| --- | --- | --- | --- | --- | --- |
| **AA1** | Decontamination activities take place in a dirty to clean workflow |  |  |  |  |
| **AA2** | Single-use items are reprocessed |  |  |  |  |

**AA3.** Design of the reprocessed medical devices

Solid, hollow Pin and box joints Lumen, tubing, tortuous paths

Porous Other (specify_____________________)

**AA4.** Material of the reprocessed medical devices

Metal Non-metal

**AB. TRANSPORT**

| **S.No.** | **Check Points** | **Yes** | **No** | **NA** | **Comments** |
| --- | --- | --- | --- | --- | --- |
| **AB1** | Medical devices are transported to the decontamination area using a rigid, durable, leak-proof container that has a tight-fitting lid |  |  |  |  |
| **AB2** | Container used for transporting medical devices is easy to clean and disinfect |  |  |  |  |

**AC. CLEANING & DISINFECTION**

**AC1.** Medical devices are cleaned before sterilization. Yes No

**AC2**. Time period between use and cleaning of medical devices _______ minutes

**AC3.** Used medical devices are soaked in or sprayed with water before cleaning to prevent drying.

Yes No

**AC3**. Personnel involved in cleaning of medical devices

Doctors Nurses HA/AHW/ANM Support staff

Other (Specify) ___________

**AC4.** Cleaning methods used

Manual Automated Both None

**AC5.** Specific procedures and solutions used for cleaning and disinfection of medical devices before sterilization

Water Water and detergent/soap Ultrasonic washers

Enzymatic cleaner Disinfectant solution Other (Specify)_______________

| **S.No.** | **Check Points** | **Yes** | **No** | **NA** | **Comments** |
| --- | --- | --- | --- | --- | --- |
| **AC6** | Cleaning is done in a separate area from where the instrument will be used (i.e., designated dirty area) |  |  |  |  |
| **AC7** | Medical devices are pre-disinfected before cleaning (e.g. with hypochlorite solution) |  |  |  |  |
| **AC8** | Following personal protective equipment are used during cleaning of used instruments |  |  |  |  |
|  | 1. Eye protection |  |  |  |  |
|  | 1. Gloves |  |  |  |  |
|  | 1. Protective clothing |  |  |  |  |
|  | 1. Facemask |  |  |  |  |
| **AC9** | Medical devices are opened/dismantled for cleaning purpose |  |  |  |  |
| **AC10** | Medical devices are submerged in water while washing them manually using a brush |  |  |  |  |
| **AC11** | For instruments with lumens, all channels are cleaned using cleaning brushes of appropriate size |  |  |  |  |
| **AC12** | Cleaning brushes are single use, disposable items |  |  |  |  |
| **AC13** | After completion of cleaning process, reusable brushes are cleaned and either high level disinfected or sterilized |  |  |  |  |
| **AC14** | Instruments are rinsed thoroughly with water after cleaning |  |  |  |  |
| **AC15** | Medical devices are dried with low-linting (disposable or reusable) towels immediately after rinsing |  |  |  |  |
| **AC16** | Enzymatic cleaner, detergent, and/or disinfectant are used according to manufacturer’s instructions |  |  |  |  |
| **AC17** | Enzymatic cleaner, detergent, and/or disinfectant are discarded according to manufacturer’s instructions |  |  |  |  |

**AD. INSPECTION**

| **S.No.** | **Check Points** | **Yes** | **No** | **NA** | **Comments** |
| --- | --- | --- | --- | --- | --- |
| **AD1** | All instruments are inspected every time after cleaning |  |  |  |  |
| **AD2** | An illuminated magnifier is used to inspect instruments |  |  |  |  |

**AE. PACKAGING**

**AE1. Sterile barrier system used**

Single wrapped/pouch

Double wrapped in wrapping material or pouches, double wrapped container or tray, reusable sterilization container according to manufacturer’s instructions

Combination of two or more systems, for example, a reusable sterilization container with an inner sterile barrier system

None

**AE2.** Wrapping material used

Paper

Cellulose/non-cellulose based non-woven wrapping materials

Cellulose/non-cellulose based woven wrapping materials

Linen

Other (specify___________)

**AE3.** Wrapping technique used

Envelope-fold wrapping technique

Square-fold wrapping technique

Other (specify____________)

| **S.No.** | **Check Points** | **Yes** | **No** | **NA** | **Comments** |
| --- | --- | --- | --- | --- | --- |
| **AE4** | Hinged devices are open and devices are disassembled (if indicated by the manufacturer) while packaging them |  |  |  |  |
| **AE5** | Packages to be sterilized are labelled with |  |  |  |  |
|  | 1. The sterilizer used |  |  |  |  |
|  | 1. The cycle or load number |  |  |  |  |
|  | 1. The date of sterilization |  |  |  |  |
|  | 1. The expiration date |  |  |  |  |

**AF. STERILIZATION (AUTOCLAVING)**

**AF1**. Personnel involved in sterilization of medical devices (autoclaving)

Doctors Nurses HA/AHW/ANM Support staff

Other (Specify) ___________

| **S.No.** | **Check Points** | **Yes** | **No** | **NA** | **Comments** |
| --- | --- | --- | --- | --- | --- |
| **AF2** | Timer is used to monitor holding period of the autoclave cycle |  |  |  |  |
| **AF3** | Holding period of the autoclave cycle starts when the pressure gauze shows the reading of required pressure (e.g.15 lbs) |  |  |  |  |
| **AF4** | The following parameters are recorded for each sterilization cycle: |  |  |  |  |
|  | 1. Cycle/load number |  |  |  |  |
|  | 1. Operator |  |  |  |  |
|  | 1. Date and Time |  |  |  |  |
|  | 1. Pressure |  |  |  |  |
|  | 1. Temperature and exposure time |  |  |  |  |
|  | 1. Holding period |  |  |  |  |
| **AF5** | Indicators used for monitoring sterilization process |  |  |  |  |
|  | 1. Autoclave tape |  |  |  |  |
|  | 1. Chemical Indicator |  |  |  |  |
|  | 1. Biological Indicator |  |  |  |  |
| **AF6** | Results for indicator recorded |  |  |  |  |
|  | 1. Autoclave tape |  |  |  |  |
|  | 1. Chemical Indicator |  |  |  |  |
|  | 1. Biological Indicator |  |  |  |  |
| **AF7** | Sterilizer physical parameters are reviewed after each run |  |  |  |  |
| **AF8** | Indicator tape is used on the outside of each wrapped package |  |  |  |  |
| **AF9** | Sterilized packs are intact and dry |  |  |  |  |

**AG. TRANSPORT AND STORAGE**

| **S.No.** | **Check Points** | **Yes** | **No** | **NA** | **Comments** |
| --- | --- | --- | --- | --- | --- |
| **AG1** | Sterilized packages are checked for integrity and compromised packages are repackaged and re-sterilized before use |  |  |  |  |
| **AG2** | Sterilized items are transported and delivered in a dry and clean container |  |  |  |  |
| **AG3** | Sterilized packages are allowed to cool down to room temperature before storage |  |  |  |  |
| **AG4** | Separate area is allocated for storage of sterilized medical devices |  |  |  |  |
| **AG5** | Sterilized packages are stored and distributed according to "the first one to enter is the first one to leave" |  |  |  |  |
| **AG6** | The area for storing sterilized packages is a well-ventilated area that provides protection against dust, moisture, insects, and temperature and humidity extremes |  |  |  |  |
